# Trait and state interoceptive abnormalities are associated with dissociation and seizure frequency in patients with functional seizures

**DOI:** 10.1111/epi.16532

**Published:** 2020-06-05

**Authors:** Akihiro Koreki, Sarah N. Garfkinel, Marco Mula, Niruj Agrawal, Sarah Cope, Talia Eilon, Cassandra Gould Van Praag, Hugo D. Critchley, Mark Edwards, Mahinda Yogarajah

**Affiliations:** ^1^ Neurosciences Research Centre St George's University of London London UK; ^2^ Department of Neuropsychiatry National Hospital Organization Shimofusa Psychiatric Medical Center Chiba Japan; ^3^ Brighton and Sussex Medical School and The Sackler Centre for Consciousness Science University of Sussex Sussex UK; ^4^ Atkinson Morley Regional Neuroscience Centre St George's Hospital London UK; ^5^ Department of Neuropsychiatry South West London and St George's Mental Health Trust London UK; ^6^ Department of Psychiatry University of Oxford Oxford UK

**Keywords:** dissociation, dissociative seizures, functional seizures, interoception, nonepileptic attack disorder, psychogenic nonepileptic seizures

## Abstract

**Objective:**

Dissociative traits represent a disturbance in selfhood that may predispose to, and trigger, functional seizures (FSs). The predictive representation and control of the internal physiological state of the body (interoception) are proposed to underpin the integrity of the sense of self (“minimal selfhood”). Therefore, discrepancies between objective and subjective aspects of interoception may relate to symptom expression in patients with FSs. Here, we tested whether individual differences in trait measures of interoception relate to dissociative symptoms, and whether state interoceptive deficits predict FS occurrence.

**Methods:**

Forty‐one participants with FSs and 30 controls completed questionnaire ratings of dissociation, and measures of (1) interoceptive accuracy (IA)—objective performance on heartbeat detection tasks; (2) trait interoceptive sensibility—subjective sensitivity to internal sensations (using the Porges Body Perception Questionnaire); and (3) state interoceptive sensibility—subjective trial‐by‐trial measures of confidence in heartbeat detection. Interoceptive trait prediction error (ITPE) was calculated from the discrepancy between IA and trait sensibility, and interoceptive state prediction error (ISPE) from the discrepancy between IA and state sensibility.

**Results:**

Patients with FSs had significantly lower IA and greater trait interoceptive sensibility than healthy controls. ITPE was the strongest predictor of dissociation after controlling for trait anxiety and depression in a regression model. ISPE correlated significantly with FS frequency after controlling for state anxiety.

**Significance:**

Patients with FSs have disturbances in interoceptive processing that predict both dissociative traits reflecting the disrupted integrity of self‐representation, and the expression of FSs. These findings provide insight into the pathophysiology of functional neurological disorder, and could lead to novel diagnostic and therapeutic approaches.

**Key Points**

- Interoception is abnormal in patients with functional seizures
- Trait interoceptive deficits are correlated with trait dissociation level
- State interoceptive deficits are correlated with seizure frequency
- These findings offer novel mechanistic insight into functional seizures

## 1 INTRODUCTION

Functional seizures (FSs), otherwise known as psychogenic nonepileptic seizures, are paroxysmal, time‐limited alterations in motor, sensory, autonomic, and/or cognitive function that superficially resemble epileptic seizures but are not caused by ictal epileptiform activity.^1^ Patients with FSs are common, and their incidence and prevalence are up to 5/100 000/y and 33/100 000, respectively.^2^ Of the 1% of the US population diagnosed with epilepsy, up to 20% actually have FSs,^3^ one in five patients presenting to a first seizure clinic have FSs,^4^ and patients with FSs have a higher mortality rate than healthy individuals.^5^ Despite this, and the finding that FSs are one of the key neuropsychiatric issues associated with epilepsy,^6^ understanding of the mechanisms underlying FSs remains poor. Dissociation is thought to be an important factor in the predisposition to and generation of the disorder. Dissociation represents a loss of, or a reduction in, the integration of psychological processes and underlying functional neural mechanisms normally amenable to volitional control. In Pierre Janet's 19th century account of hysteria,^7^ FSs are conceptualized as intrusive sensorimotor flashbacks with psychological fragmentation, when mental functions including memories of traumatic events are separated or “dissociated” from consciousness. Here, FSs are considered a defensive process to manage otherwise overwhelming feelings, experiences, or stress.^7^ In this context, FSs represent a form of “somatoform” dissociation where dissociative symptoms phenomenologically involve the body^8^ and reflect the disintegration of neural systems normally amenable to deliberate control.^9^ This can be measured using the Somatoform Dissociation Questionnaire (SDQ‐20).^10^ A variant of this account describes FSs as altered states of consciousness similar to panic attacks, but in which the subjective fear component is dissociated from awareness. Here, dissociative symptoms phenomenologically involve psychological variables,^8^ and the dissociation reflects a state of “detachment” encompassing depersonalization.^11^ This form of “psychological” dissociation can be measured using the depersonalization subscale of the Multiscale Dissociation Inventory (MDI).^12^

Such psychological‐level accounts are poorly integrated with advances in neurobiological understanding of brain function. Theoretical neuroscience has recently undergone a paradigmatic shift. The brain is no longer thought of as a passive processor of sensory information from the external world. Instead, it is now typically considered an active organ of inference, generating predictions and hypotheses about the causes of its sensations, which in turn give rise to perception and action.^13^ Such “Bayesian” or predictive processing models extend to the sensation and perception of the body “from within,” otherwise known as interoception.^14^ Interoception refers collectively to the body‐to‐brain axis of signals originating from the internal body and visceral organs. In an extended interoceptive model, the sense of body ownership or “presence” (minimal selfhood), is determined by minimization of the discrepancy between top‐down predictions (priors) of the interoceptive state of the body and bottom‐up incoming interoceptive signals.^15^ Conversely, this model maintains that disorders of presence or body ownership, such as dissociation, result from pathologically imprecise interoceptive predictive signals and increased discrepancy between top‐down and bottom‐up signals.^15^ This model has been supported by studies demonstrating interoceptive deficits in patients with primary dissociative disorders.^16^

There are distinct channels of interoception, but the cardiovascular system is most commonly studied. Individual interoceptive differences manifest across different dimensions of interoception.^17^ Interoceptive accuracy refers to the objective measure of how well an individual performs on interoceptive tasks. In predictive coding terms, it represents the ability to prioritize interoception over other sensory modalities, and thus adjust the relative precision (weighting) of interoceptive priors and prediction errors (that part of incoming interoceptive sensation not accounted for by priors), thereby minimizing the discrepancy between top‐down and bottom‐up signaling.^18^ This is dissociable from interoceptive sensibility, which refers to the subjective impression of an individual about his/her sensitivity to internal signals. In predictive coding terms, this represents the precision (weighting) afforded to subjective belief/self‐model of general interoceptive ability. A third dimension, describes the discrepancy or mismatch (prediction errors) between these measures at both a state and trait level.

We sought to assess interoceptive function in patients with FSs, and to characterize the relationship between interoceptive errors, dissociation, and seizures. We hypothesized that patients with FSs would have poorer interoceptive accuracy compared to healthy controls. We also expected that the greater the discrepancy between top‐down and bottom‐up measures of interoception at a trait and state level, the greater the levels of trait dissociation and seizure frequency, respectively, in patients with FSs.

## 2 MATERIALS AND METHODS

### 2.1 Participants

Patients with a diagnosis of FSs attending the outpatient department at Atkinson Morley Regional Neuroscience Unit at St George's University Hospital, London between January 2018 and January 2019 were sequentially included in this study. The diagnosis of FSs was made according to International League Against Epilepsy diagnostic criteria by at least two clinicians experienced in the diagnosis of epilepsy, and were documented (n = 21), clinically established (n = 11), or probable (n = 9) cases.^1^ A group of age/gender‐matched healthy controls were recruited by way of advertisements placed at St George's University/Hospital.

Exclusion criteria for both groups included age < 18 years, language difficulties, learning disability, and concurrent chronic neurological/medical conditions, or administration of medications with direct cardiac effects such as beta‐blockers.

All participants gave informed consent for the study. The ethics committee of Fulham, London approved the study protocol, and the study was also approved by the Health Research Authority (IRAS 231863, REC 18/LO/0328). All participants provided informed consent.

### 2.2 Stimuli

Interoceptive accuracy was judged by the participants' ability to detect their own heartbeats using a heartbeat tracking task (HTT)^19^ and a heartbeat discrimination task (HDT).^20^ As a control for the heartbeat counting task, participants also completed a time tracking task (TTT). These tasks are described further in the Supporting Information. On each interoceptive trial, participants completed a visual analogue scale (VAS) to signal confidence in their interoceptive decision. This provided one subjective measure of interoception (sensibility), used to compute metacognitive interoceptive awareness/interoceptive state prediction error (ISPE; see below). See Supporting Information for experimental procedures.

Trait interoceptive sensibility was determined using the awareness section of the Porges Body Perception Questionnaire (BPQ).^21^ This subscale covers distinct bodily sensations; participants indicated their awareness of each sensation using a 5‐point scale ranging from “never” to “always.” Given that the BPQ asks participants to rate how frequently they perceive/detect bodily sensations, this subjective measure in part reflects the participants' belief in their own interoceptive ability, regardless of objectively determined interoceptive accuracy.

Anxiety was assessed using the Spielberger State/Trait Anxiety Inventory (STAI),^22^ and depression with the Beck Depression Inventory (BDI).^23^ Dissociative symptoms were assessed using two questionnaires: the SDQ‐20^10^ and the MDI.^12^ The MDI is the only standardized and normed measure of dissociative responses, and is based on a multidimensional construct, unlike the more widely used but unstandardized Dissociative Experiences Scale. The depersonalization subscore of the MDI (MDI‐DP)^12^ was used to assess depersonalization, rather than a dedicated depersonalization inventory (eg, Cambridge Depersonalisation Scale) to assess the specificity of the relationship between interoception and depersonalization, compared to other dimensions of dissociation.

### 2.3 Data analysis

#### 2.3.1 Interoceptive accuracy and sensibility

Methods for deriving interoceptive accuracy and sensibility have been described previously^24^ and are outlined in the Supporting Information.

#### 2.3.2 Interoceptive awareness/ISPE

ISPE represents the trial‐by‐trial correspondence between performance accuracy (correct synchronous/asynchronous decisions) and confidence (trial‐by‐trial VAS rating) during performance of the heartbeat discrimination. A receiver operating characteristic (ROC) analysis was performed to compute the diagnostic significance of confidence for accuracy on a trial‐by‐trial basis.^24^ Correct identification of whether the tones were synchronous or asynchronous with the heart served as the state variable; rated confidence served as the test variable. Area under the ROC curve denoted the degree to which confidence is predictive of accuracy.^24^ The inverse of this measure of metacognitive interoceptive awareness can be considered ISPE. Individuals have low ISPE or high interoceptive awareness when they know they performed well when they did actually perform well or know they performed badly when they actually performed badly.

#### 2.3.3 Interoceptive trait prediction error

The interoceptive trait prediction error (ITPE) was operationalized as the difference between objective interoceptive accuracy and subjective interoceptive sensibility.^17^ For each interoceptive accuracy and sensibility variable (heartbeat tracking score, heartbeat detection score, and awareness subsection of the BPQ), scores were converted to standardized *Z* values. On a within‐participants basis, ITPE values were calculated as the difference between interoceptive sensibility and interoceptive accuracy. ITPEs were calculated separately using accuracy scores from each task (heartbeat tracking [ITPE_T_] and heartbeat discrimination [ITPE_D_]), using in each case a sensibility score provided by the awareness section of the BPQ. The larger and more positive the value of ITPE, the greater the interoceptive error. Individuals have a high ITPE when they think they are good at detecting interoceptive signals generally, but are poor on testing.

### 2.4 Statistical analyses

Group differences in dimensions of interoception (interoceptive accuracy, interoceptive sensibility, ISPE, ITPE), trait/state anxiety, SDQ‐20, BDI, and MDI‐DP were determined using independent *t* tests. Where Levene test for the equality of variances was found to be violated, equal variances were not assumed, and *df*, *t*, and significance values were adjusted accordingly using the Welch *t* test. Pearson *r* was used to assess the relationships between dissociation (SDQ‐20 and MDI‐DP) and ITPE. We also assessed correlations between ITPE and other domains of the MDI to determine the specificity of the relationship of ITPE and MDI‐DP. Preliminary analyses were carried out to confirm the assumptions of linearity, normality, and homoscedasticity.

A regression analysis in patients was performed with dissociation (SDQ‐20 or MDI‐DP) as the dependent variable. All predictor variables were included in the model (interoceptive accuracy, interoceptive sensibility, ITPE, trait STAI, BDI). Heartbeat discrimination served as the measure for interoceptive accuracy, and ITPE_T_ was calculated as described above. When accuracy on heartbeat tracking and ITPE_D_ were instead entered into the regression model, the significant contribution of the interoceptive error to dissociation measures was maintained except for SDQ‐20, where interoceptive heartbeat tracking accuracy rather than ITPE_D_ was predictive. Preliminary analyses were conducted to ensure no violation of the assumptions of normality of residuals, linearity, multicollinearity, and homoscedasticity.

Seizure severity was defined as the reported frequency (weekly) of FSs. When assessing the correlation between interoceptive measures and seizure severity, there were several outliers with respect to seizure frequency, and the assumptions of normality and linearity were broken. As a result of this, a partial Spearman rank correlation test was used, while correcting for state STAI scores.

All reported analyses are two‐tailed.

### 2.5 Effect sizes and multiple testing correction

Cohen *d* was used as an effect size measure for comparisons between groups. False discovery rate (FDR) control using the Benjamini and Hochberg method was used to correct *P* values and control error rates arising from multiple statistical testing.^25^

## 3 RESULTS

### 3.1 Group comparisons

#### 3.1.1 Participant demographics and behavioral measures

Patients with FSs were matched for key demographics with no significant group differences for sex, age, or heart rate variability. However, controls reported significantly smaller SDQ‐20 (*P* < .001*,* *d* = −2.59), trait anxiety (*P* < .001*,* *d* = −1.54), state anxiety (*P* < .001*,* *d* = −1.61), BDI (*P* < .001*,* *d* = −1.84), and MDI‐DP (*P* < .001*,* *d* = −2.15) scores than patients (Table 1). The mean duration of FSs in patients was 5.4 ± 0.79 years.

**TABLE 1:** Participant demographics and behavioral measures

	Control (SEM)	Functional seizures (SEM)
Sex, males/females	3/27	2/39
Age, y	32 (2)	32 (2)
Trait anxiety^a^	30 (1)	46 (2)
State anxiety^a^	40 (1)	59 (2)
BDI^a^	3 (1)	21 (2)
SDQ‐20^a^	21 (0)	38 (2)
MDI‐DP^a^	5 (1)	11 (1)
Heart rate variability, RMSSD, ms	77 (9)	59 (8)

Abbreviations: BDI, Beck Depression Inventory; MDI‐DP, depersonalization subscale of Multiscale Dissociation Inventory; RMSSD, root mean square of successive differences; SDQ‐20, Somatoform Dissociation Questionnaire; SEM, standard error of the mean.

^a^ Significant differences at *P* < .05.

#### 3.1.2 Interoceptive accuracy and sensibility

Patients with FSs were objectively impaired in interoceptive accuracy, as reflected by a significantly reduced performance on the HTT (*P* < .001, *d* = 1.12; Table 2). Although the accuracy of patients was also impaired during the HDT (proportion correct = 0.52, standard error of the mean [SEM] = 0.218) relative to controls (proportion correct = 0.56, standard error of the mean = 0.261), this difference did not meet threshold significance (Table 2). However, a one‐sample *t* test showed that whereas patients performed no better than chance (50%), controls did perform significantly better than chance (*P* = .025). There was no significant difference in the performance on the TTT between patients and controls (Table 2).

**TABLE 2:** Interoceptive accuracy and sensibility measures

	Control (SEM)	Functional seizures (SEM)
HTT accuracy^a^	0.71 (0.03)	0.40 (0.07)
HDT accuracy	56.17 (2.61)	52.5 (2.18)
TTT accuracy	0.75 (0.03)	0.71 (0.04)
Sensibility^a^	43.33 (1.72)	54.63 (1.41)

Abbreviations: HDT, heartbeat discrimination task; HTT, heartbeat tracking task; SEM, standard error of the mean; TTT, time tracking task.

^a^ Significant differences at *P* < .05.

#### 3.1.3 Interoceptive sensibility

Patients with FSs scored significantly higher on the awareness subscale of the Porges BPQ (Table 2). This indicates enhanced subjective interoceptive sensibility. Given that the BPQ asks people to rate how frequently they perceive/detect bodily sensations, this can be interpreted as an increased belief in interoceptive aptitude, or the precision (weighting) afforded to subjective belief/self‐model of general interoceptive ability relative to control participants (*P* < .001*,* *d* = −1.14).

#### 3.1.4 Interoceptive awareness

There was no difference in state interoceptive awareness between the patients with FSs and controls for the HDT.

#### 3.1.5 Interceptive trait prediction error

The ITPE, defined as the difference between subjective sensibility and objective accuracy for the HTT (ITPE_T_) and the HDT (ITPE_D_), tended to be positive for patients with FSs (mean [SEM] = 0.76 [0.26], 0.52 [0.20]). Together, these ITPE scores signal that participants with FSs were likely to score higher on subjective sensibility relative to the two objective tests of interoceptive accuracy. In contrast, the reverse trend was displayed by control participants, who tended to display greater accuracy values for both the tracking and discrimination tasks relative to subjective sensibility, resulting in lower scores for both ITPE_T_ (−1.04 [0.13]) and ITPE_D_ (−0.73 [0.23]). Moreover, the values for ITPE_T_ and ITPE_D_ both significantly differed between the two groups (*P* < .001*,* *d* = −1.61 and *P* < .001*,* *d* = −1.02, respectively).

All significant *P* values for the group comparisons survived FDR correction at a threshold of .05.

### 3.2 Relationship to dissociation

The relationship between interoception and dissociation was examined only in the patients with FSs, and not in controls.

**FIGURE 1:**
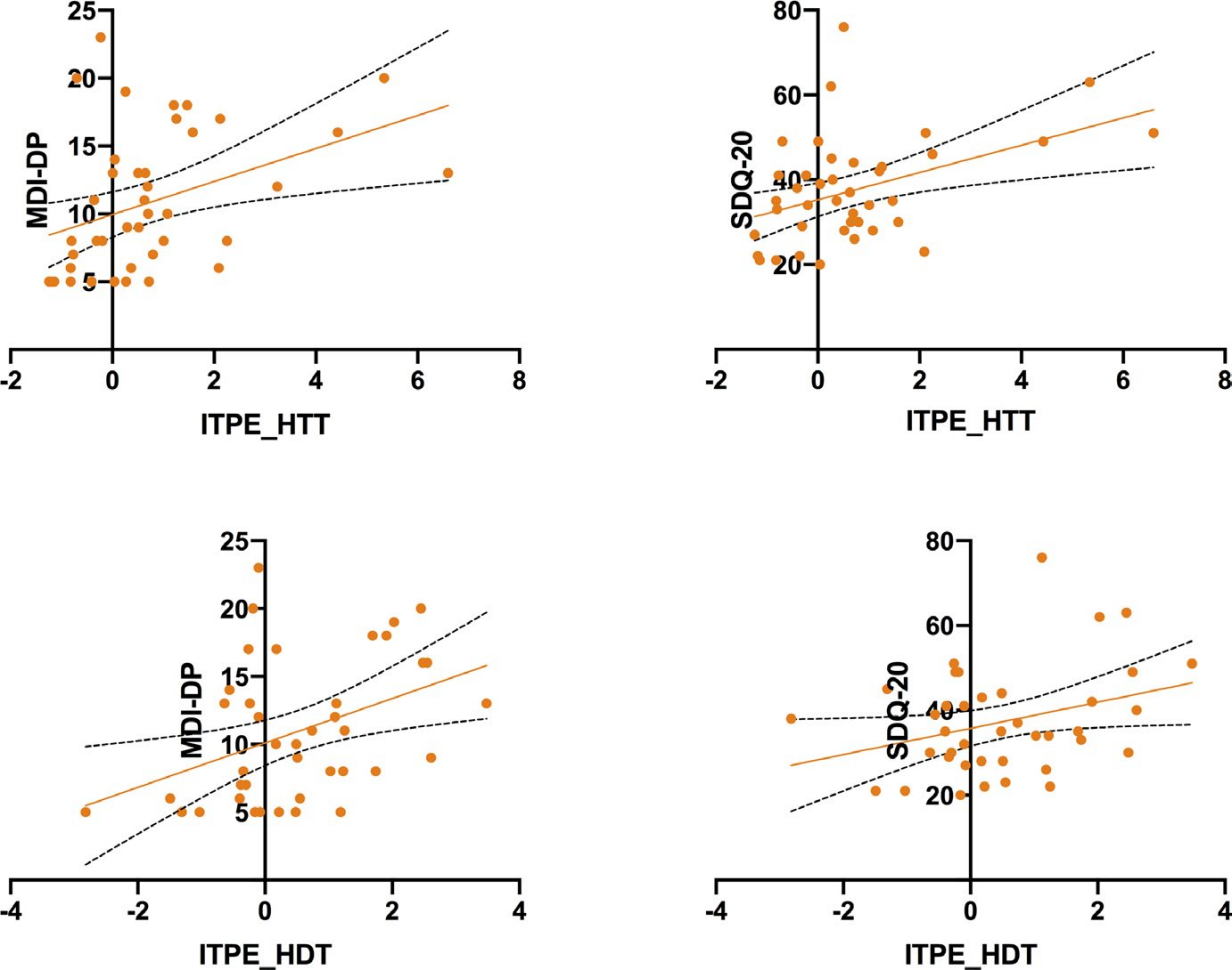
Interoceptive trait prediction error (ITPE) derived from the heartbeat tacking task (HTT) and heartbeat discrimination task (HDT) plotted against Somatoform Dissociation Questionnaire (SDQ‐20) and depersonalization subscore of the Multiscale Dissociation Inventory (MDI‐DP) scores

Addressing our central hypothesis, we tested for a correlation between dissociation measures and ITPE (Figure 1). This was examined separately for HDT and HTT accuracy. Throughout, a positive relationship emerged; ITPE_T_ correlated with both SDQ‐20 (*r* = .429, *P* = .006) and MDI‐DP (*r* = .398, *P* = .01) scores. Similarly, ITPE_D_ positively predicted both and MDI‐DP (*r* = .404, *P* = .01) and SDQ‐20 (*r* = .320, *P* = .046) scores. There was no significant correlation between trait prediction errors derived from the TTT and the SDQ‐20 or MDI‐DP scores. All significant *P* values survived FDR correction at a threshold of .05.

To test the specificity of the relationship between ITPE and MDI‐DP and SDQ‐20, we repeated the above two analyses but included all the subscales of the MDI‐DP (disengagement, depersonalization, derealization, emotional constriction, memory disturbance, and identity dissociation), and the SDQ‐20. In these analyses, the only significant correlation to survive FDR correction at a threshold of .05 in addition to those reported above was between ITPE_T_ and the identity subscale (*r* = .369, *P* = .018). None of the correlations between ITPE_D_ and dissociation indices survived FDR correction at a threshold of .05.

To dissect the relative importance of interoceptive accuracy, interoceptive sensibility, and ITPE to dissociation, all variables were entered into a multiple regression model. Trait anxiety and depression scores were also included in the regression analysis because of their potential confounding effects. In both models where SDQ‐20 and MDI‐DP were the dependent variables, ITPE_T_ alongside BDI was a significant predictor (Table 3). All significant *P* values survived FDR correction at a threshold of *P* ≤ .05.

**TABLE 3:** Regression model of ITPE against dissociation measures in patients

	*B*	SE *B*	*β*	*P*
Model 1: MDI‐DP (*R* ^2^ = .30)				
Constant	6.99	1.34		<.001
BDI	0.15	0.05	0.38	.010
ITPE	1.08	0.43	0.35	.015
Model 2: SDQ‐20 (*R* ^2^ = .27)				
Constant	29.36	3.33		.001
ITPE	2.91	1.06	0.39	.009
BDI	0.28	0.13	0.30	.041

Abbreviations: BDI, Beck Depression Inventory; ITPE, interoceptive trait prediction error; MDI‐DP, depersonalization subscale of Multiscale Dissociation Inventory; SDQ‐20, Somatoform Dissociation Questionnaire; SE, standard error.

### 3.3 Relationship to clinical severity

**FIGURE 2:**
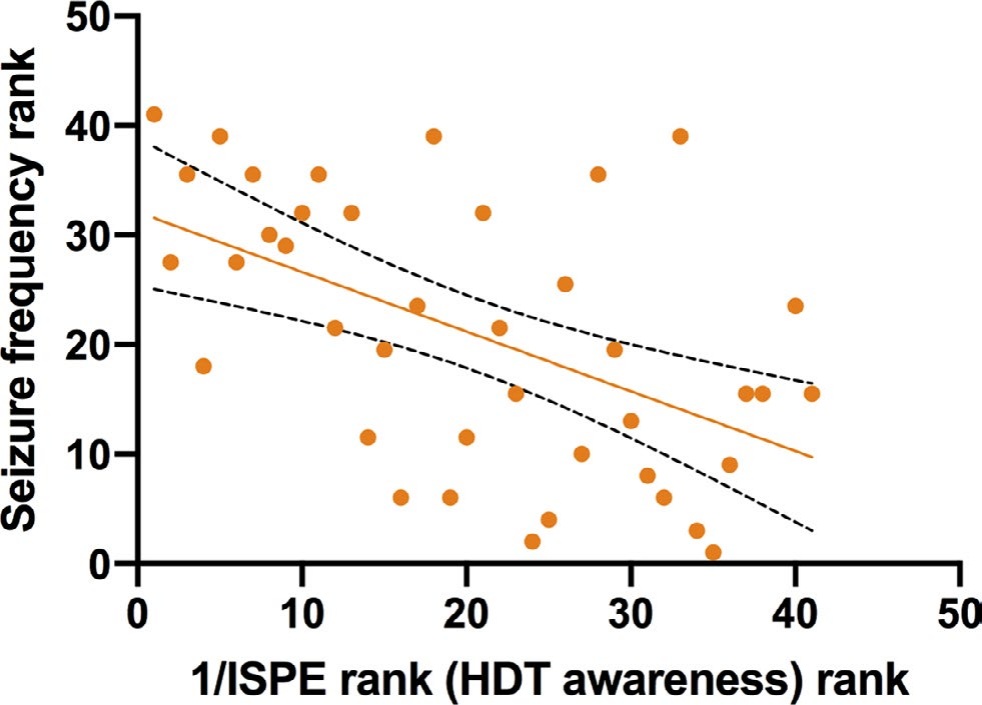
Seizure frequency rank plotted against 1/interoceptive state prediction error (ISPE) rank. Note that the bigger the value for seizure frequency rank, the smaller seizure frequency in the patient population. The bigger the 1/ISPE rank, the bigger the ISPE value. 1/ISPE is equivalent to heartbeat discrimination task (HDT) awareness

There was no significant correlation between ITPE_T_ or ITPE_D_ and seizure frequency. However, there was a significant negative correlation between interoceptive awareness or ISPE and seizure frequency after correcting for state anxiety levels (*r*_s_ = −.529, *P* = .001; Figure 2). This correlation survived FDR correction at a threshold of .05.

## 4 DISCUSSION

This is the first study investigating the relationship between interoception and dissociative symptoms in patients with FSs. We confirmed that patients with FSs have significantly higher levels of somatoform dissociation and depersonalization compared with healthy controls. Here, we show that patients with FSs have significantly greater interoceptive sensibility, but lower interoceptive accuracy compared with healthy controls. The bigger the discrepancy between a patient's prior beliefs about their general ability to detect their internal bodily signals, and their objective accuracy in judging interoceptive signals (heartbeat detection), the greater their reported levels of dissociation as measured by the MDI‐DP and SDQ‐20. Moreover, the bigger the discrepancy between a patient's trial‐by‐trial confidence in detecting their heartbeat, and their objective interoceptive accuracy, the greater the frequency of their seizures. These findings represent an important biological window into the mechanisms underlying FSs.

### 4.1 Dissociation and FSs

We have demonstrated that dissociative traits are more common in patients with FSs, compared with healthy individuals. Patients with FSs report higher rates of depersonalization, as measured by the MDI‐DP, and higher rates of somatoform dissociation or somatic phenomena, as measured by the SDQ‐20. These findings are similar to those reported by the only other study to use both the MDI and SDQ‐20 in patients with FSs.^26^ In this study, SDQ‐20 scores were elevated in patients with FSs, and were a robust predictor of FSs. This study also demonstrated that patients who experience more dissociation during FSs experience higher levels of trait depersonalization as measured by the MDI‐DP.

### 4.2 Interoception and FSs

To our knowledge, there is only one published study that has previously explored interoception in patients with FSs. This study also included patients with functional movement disorders.^27^ No suprathreshold differences were reported in measures of interoceptive accuracy in a group of 20 patients with FSs compared to 20 healthy controls. However, this study also found no differences in interoceptive accuracy in patients with functional movement disorders compared to controls. This contrasted with findings reported by the same group reporting decreased cardiac interoceptive accuracy in patients with functional movement disorders relative to controls.^28^ Our finding of reduced interoceptive accuracy in patients with FSs reported here may therefore be a reflection of our larger sample size, and hence greater power to detect group differences.

The increased discrepancy between subjective overestimation of interoceptive perception and objective accuracy observed in patients with FSs compared to healthy controls has been reported in other studies of bodily awareness in patients with functional neurological disorders. These studies highlight mismatch between subjective and objective symptom reports in patients. Patients with functional tremor, in diary recordings, report that the tremor is present most of the time, despite a dominant absence of objective measurement of tremor recorded using wrist‐worn accelerometers.^29^ Similarly, patients with functional movement disorders tended to overestimate their symptom severity on subjective symptom ratings, relative to their ratings when watching videos of themselves and their symptoms.^30^ Finally, patients with functional movement disorders undertaking the Trier Social Stress Test show a discrepancy between biochemical measures of stress and perceived stress levels.^31^

### 4.3 Interoception, dissociation, and FSs

The experience of “selfhood” can be thought of as a continuous and integrated process that includes body ownership, intention, and agency, and the experience of being a continuous self and social self.^32^ Dissociation can therefore be conceptualized as the perturbation of selfhood, and specific subtypes of dissociation may reflect breakdown of particular aspects of selfhood. Interoception is proposed to play a direct role in structuring experiences of “being and having a body.”^33^ Dissociation is therefore plausibly related, at a neurobiological level, to abnormalities in interoception. In support of this, the two phenomena share common anatomical substrates.^34^ Patients with subclinical dissociative identity disorder accompanied by symptoms of dissociation show impaired interoceptive accuracy on HTTs.^16^ Similar findings are apparent in patients with symptoms of depersonalization,^35^ and in patients with somatoform disorders there is a negative correlation between symptom load and interoceptive accuracy.^36^ Our findings of lower levels of interoceptive accuracy, and higher rates of dissociation in patients with FSs therefore extend these earlier observations.

Active inference is a theory about how the brain makes sense of the world and body in which it is embedded. It formalizes the idea that the brain must discover information about the likely causes of sensory signals (ie, perception) without direct access to these causes, using only information in the sensory signals themselves. This is accomplished by probabilistic inference on the causes of sensory signals, computed according to Bayesian principles. In practice, this means estimating the probable causes of sensory data, given the observed data and prior “beliefs” about probable causes.^37^ A key component of predictive processing is the mismatch or error between prediction and signal. Predictive coding models describe counter‐flowing top‐down prediction/expectation signals and bottom‐up prediction error signals. Successful perception, cognition, and action are associated with successful suppression (“explaining away”) of prediction error. Predictive coding models have also been applied to interoception in, for example, studies of emotion,^38^ depression, and anxiety.^39^ It is also proposed that “presence” is the result of successful suppression by top‐down predictions of informative interoceptive signals evoked (directly) by autonomic control signals and (indirectly) by bodily responses to afferent sensory signals. Thus, disorders of presence, including dissociation, could conceivably result from pathologically imprecise interoceptive predictive signals.^15^ Our findings lend support to this model of the mechanism underlying dissociative experiences. ITPE is a metric of the divergence between objective sensory sensitivity to interoceptive information and the subjective longer‐term belief/self‐model of general interoceptive ability. It can also be conceived as the discrepancy between the weighted average of higher‐level interoceptive predictions and the ability to adjust or update these predictions in the light of new sensory information. Although adaptive, normal functioning relies on error signals to adjust expectations and perception, the persistence of error signals due to inadequate adjustment leads to dysfunctional processing.^40^ ITPE scores were larger in participants with FSs, reflecting that they were likely to score higher on subjective sensibility relative to the two objective tests of interoceptive accuracy compared to controls. This can be interpreted as a tendency in participants with FSs to have higher‐level interoceptive predictions held with undue belief or precision (weighting), and an inability to update these beliefs based on incoming interoceptive information due to its imprecision. This error term correlated significantly and specifically with measures of dissociation derived from the MDI‐DP and SDQ‐20 scores. Importantly, ITPE, rather than interoceptive sensibility, accuracy, and anxiety, was a significant predictor of dissociation, alongside depression.

This interpretation of trait dissociation in patients with FSs is in keeping with contemporary models of functional disorders, where symptoms are proposed to represent an “inferential leap” in which the brain interprets information from the body in the light of expectations or predictions held with undue precision (weighting) given past experience.^41^ Diminished interoceptive awareness, or the discrepancy between interoceptive accuracy and a participant's trial‐by‐trial subjective opinion of their interoceptive accuracy, is an index of one prediction error, concerning interoceptive state (ISPE). Prediction errors in this setting refer to moment‐to‐moment discrepancies between expected and actual interoceptive signals, rather than trait‐based differences between objective and subjective performance (as indexed by ITPE). Given that patients with FSs typically experience dissociative and somatic symptoms such as palpitations, sweating, shortness of breath, and pins and needles before and during their FSs,^42^ it is plausible that ISPE or interoceptive awareness based on cardiac channels, rather than trait‐based measures (ITPE), should correlate with a patient's seizure frequency. That is, the more prone a patient is to specifically misinterpret their cardiac physiological signals, the more frequent are their FSs.

### 4.4 Limitations

We did not include all potential confounders when modeling the relationship between interoception, dissociation, and seizure severity, including heart rate variability,^16^ depression, educational attainment, and body mass index.^43^ A larger number of participants is required to power the statistical assessment of such confounding covariates. However, we did include depression and anxiety in our statistical models, and we found no group differences in heart rate variability. Most patients with FSs were medicated, which could have affected the reported results. Nevertheless, we excluded patients taking cardiotropic medications, and previous studies report no differences in heartbeat perception between medicated and nonmedicated depressed patients.^44^, ^45^ Information about psychiatric comorbidities identified during structured interviews was not available in all patients, and this may be a confounding factor. However, all patients had depression and anxiety assessed using inventories, and these were incorporated where relevant. There is evidence suggesting that performance on the HTT may be colored by noninteroceptive factors, notably knowledge concerning own heart rate and time estimation ability.^46^, ^47^ In mitigation, we included a time tracking control task, and found no group differences in this task, and no correlations between trait error measurements and dissociative measurements. We also note our study was cross‐sectional, limiting both inferences about causal mechanisms, and seizure frequency accuracy. Finally, future studies should also explore the role interoception might play in other established biological models of dissociation, which include the over‐ and undermodulation of affect.^48^

## 5 CONCLUSIONS

This study provides the first evidence for abnormalities of interoception in patients with FSs. These abnormalities correlate with the levels of trait dissociation in patients and the frequency of their seizures. They provide a biological window into the nature of FSs, and represent a potential transdiagnostic biomarker underlying FSs and their comorbidities, as well as the basis for novel therapeutic approaches to the management of patients with FSs.

## CONFLICT OF INTEREST

None of the authors has any conflict of interest to disclose. We confirm that we have read the Journal's position on issues involved in ethical publication and affirm that this report is consistent with those guidelines.

## Supporting information

Supplementary Material
