# Supplementary material for: Trait and state interoceptive abnormalities are associated with dissociation and seizure frequency in patients with functional seizures
Source: Epilepsia. 2020 Jun 5;61(6):1156–65. doi: 10.1111/epi.16532 (PMC7737228; doi:10.1111/epi.16532)
Supplement: Supplementary file 1 — Supplementary Material [file EPI-61-1156-s001.docx]

**Supplementary methods**

**Heartbeat tracking and discrimination tasks**

During the heartbeat-tracking task, participants’ heartbeats were monitored via a medical grade pulse oximeter (Nonin Xpod 3012LP) with a ‘soft’ sensor (to prevent finger-pulse sensation) mounting attached to their index finger. Participants were required to count their heartbeats during six randomized time windows of varying length (25, 30, 35, 40, 45 and 50 s) and, at the end of the trial, to report the number of heartbeats detected to the experimenter. For the heartbeat discrimination task, each trial consisted of ten tones presented at 440 Hz and having 100 ms duration, which were triggered by the heartbeat. Under the asynchronous condition, a delay of 300 ms was inserted, adjusting for the average delay (~250 ms) between the R-wave and the arrival of the pressure wave at the finger^1^. Tones were thus presented at 250 ms or 550 ms after the R-wave, which correspond to maximum and minimum synchronicity judgements respectively^2^. At the end of each trial, participants signalled whether they believed the tones to be synchronous or asynchronous with their heartbeats.

**Experimental procedures**

Following informed consent, before each trial participants completed the questionnaires, and had an initial 6-minute period of continuous beat-to-beat blood pressure and pulse monitoring using a Finometer Pro (Finapres Medical Systems) prior to interoceptive testing. From this period, data measurements were exported for analysis in order to derive heart rate variability (HRV) measurements (Kubios HRV).

All participants then performed the cardiac perception (interoceptive) tasks. To prevent the temporal timing of tones priming participants towards their own heart rate, the HDT was always presented after the HTT. Just prior to starting, participants were asked to sit quietly and told to focus internally, in order to try to feel their own heart beating. For the heartbeat tracking task, participants were given the following instructions: ‘Without manually checking, can you silently count each heartbeat you feel in your body from the time you hear *start* to when you hear *stop*’. This was repeated a total of 6 times using a variety of randomized trial lengths (25, 30, 35, 40, 45 and 50 s). During the HDT, each participant was told: ‘You will hear ten tones. Please can you tell me if the tones are in or out of sync with your heartbeat’. This was repeated for a total of 20 times Finally, as a control for the heartbeat counting task, participants also completed a time tracking task (TTT). Here they were provided with the following instructions: ‘You will hear a start and stop signal. Please estimate the number of seconds that have elapsed between these signals.’ This was repeated a total of six times using a variety of randomized trial lengths (25, 30, 35, 40, 45 and 50 s). On each interoceptive trial, participants completed a visual analogue scale (VAS) to signal confidence in their interoceptive decision.

**Data Analysis**

**Interoceptive accuracy (IA)**

To derive measures for interoceptive accuracy, heartbeat tracking scores were calculated on a trial-by-trial basis based upon the ratio of perceived to actual heartbeats: 1 – (nbeats_real_ – nbeats_reported_)/((nbeats_real_ + nbeats_reported_)/2)^3^ and these were averaged to form a mean heartbeat tracking score. This measure calculates interoceptive accuracy independent of the amount of heartbeats in the trial by normalising the absolute error in perceived heartbeats as a function of the overall number of heartbeats.

Interoceptive accuracy for the heartbeat discrimination task was assessed as a ratio of correct to incorrect synchronicity judgments.

**Interoceptive sensibility**

To assess interoceptive sensibility, total score on the awareness section of Porge’s Body Perception Questionnaire (BPQ) was calculated for each participant^3^.

1. Payne RA, Symeonides CN, Webb DJ, et al. Pulse transit time measured from the ECG: an unreliable marker of beat-to-beat blood pressure. J Appl Physiol. 2006; 100(1):136–41.

2. Wiens S, Palmer SN. Quadratic trend analysis and heartbeat detection. Biol Psychol. 2001; 58(2):159–75.

3. Garfinkel SN, Seth AK, Barrett AB, et al. Knowing your own heart: distinguishing interoceptive accuracy from interoceptive awareness. Biol Psychol. 2015; 104:65–74.

**Table of Acronyms**

| **Acronym** | **Meaning** |
| --- | --- |
| BDI | Beck depression inventory |
| BPQ | Body perception questionnaire |
| DES | Dissociative experiences scale |
| FDR | False discovery rate |
| FS | Functional seizure |
| HDT | Heartbeat discrimination task |
| HRV | Heart rate variability |
| HTT | Heartbeat tracking task |
| IAcc | Interoceptive accuracy |
| IS | Interoceptive sensibility |
| ISPE | Interoceptive state prediction error |
| ITPE | Interoceptive trait prediction error |
| ITPE_D_ | Interoceptive trait prediction error based on heartbeat discrimination task |
| ITPE_T_ | Interoceptive trait prediction error based on heartbeat tracking task |
| MDI (DP) | Multi-scale dissociation inventory (depersonalisation sub-score) |
| PNES | Psychogenic non-epileptic seizures |
| ROC | Receiver operator characteristic |
| SDQ-20 | Somatoform dissociation questionnaire |
| STAI | State and trait anxiety inventory |
| TTT | Time tracking task |
| VAS | Visual analogue scale |
